# Multi-omics insights into the positive role of strigolactone perception in barley drought response

**DOI:** 10.1186/s12870-023-04450-1

**Published:** 2023-09-22

**Authors:** Agata Daszkowska-Golec, Devang Mehta, R. Glen Uhrig, Agnieszka Brąszewska, Ondrej Novak, Irene M. Fontana, Michael Melzer, Tomasz Płociniczak, Marek Marzec

**Affiliations:** 1 Institute of Biology, Biotechnology and Environmental Protection, Faculty of Natural Sciences, University of Silesia in Katowice, Jagiellonska 28, 40-032 Katowice, Poland; 2 Department of Biological Sciences, University of Alberta, 11455 Saskatchewan Drive, Edmonton, AB T6G 2E9 Canada; 3 Laboratory of Growth Regulators, Faculty of Science, Palacký University & Institute of Experimental Botany, The Czech Academy of Sciences, Olomouc, Czech Republic; 4 Leibniz Institute of Plant Genetics and Crop Plant Research (IPK), Gatersleben, Seeland, 06466 Gatersleben, OT Germany

**Keywords:** Abscisic acid, Barley (*Hordeum vulgare*), Drought, Phytohormone, Proteome, Strigolactone, Transcriptome

## Abstract

**Background:**

Drought is a major environmental stress that affects crop productivity worldwide. Although previous research demonstrated links between strigolactones (SLs) and drought, here we used barley (*Hordeum vulgare*) SL-insensitive mutant *hvd14* (*dwarf14*) to scrutinize the SL-dependent mechanisms associated with water deficit response.

**Results:**

We have employed a combination of transcriptomics, proteomics, phytohormonomics analyses, and physiological data to unravel differences between wild-type and *hvd14* plants under drought. Our research revealed that drought sensitivity of *hvd14* is related to weaker induction of abscisic acid-responsive genes/proteins, lower jasmonic acid content, higher reactive oxygen species content, and lower wax biosynthetic and deposition mechanisms than wild-type plants. In addition, we identified a set of transcription factors (TFs) that are exclusively drought-induced in the wild-type barley.

**Conclusions:**

Critically, we resolved a comprehensive series of interactions between the drought-induced barley transcriptome and proteome responses, allowing us to understand the profound effects of SLs in alleviating water-limiting conditions. Several new avenues have opened for developing barley more resilient to drought through the information provided. Moreover, our study contributes to a better understanding of the complex interplay between genes, proteins, and hormones in response to drought, and underscores the importance of a multidisciplinary approach to studying plant stress response mechanisms.

**Supplementary Information:**

The online version contains supplementary material available at 10.1186/s12870-023-04450-1.

## Background

Plants have developed diverse and efficient strategies to survive periods of drought [1], many of which are controlled by phytohormones, including strigolactones (SLs). SLs are a group of carotenoid derivatives regulating the various aspects of plant growth and development, mainly related to the shoot and root architecture [2]. SLs are recognized and bound by the SL receptor D14 (DWARF14), which possesses an enzymatic activity [3]. Interaction with SL changes the conformation state of the receptor, allowing it to bind D14 along with the MAX2 (MORE AXILLARY GROWTH2) protein. MAX2 is an F-box leucine-rich repeat protein [4, 5], part of the Skp1 (S-Phase Kinase Associated Protein1)-Cullin-F-box (SCF) complex. The D14-SCF^MAX2^ complex then functions to polyubiquitinate SL transcriptional repressors, resulting in their degradation, followed by the transcription activation of the SL-dependent genes [6, 7].

First indications that SLs may be involved in plant drought response were published in 2014, based on the analysis of Arabidopsis (*Arabidopsis thaliana*) mutants, affected in SL biosynthesis and signaling [8, 9]. Both reports indicated that Arabidopsis SL signaling mutant *max2* is more sensitive to drought when compared to the wild-type (WT) Columbia-0 (Col-0) plants [8, 9]. Here, it was shown that application of the synthetic SL analog rac-GR24 increases drought resistance in Arabidopsis [9], which was later also resolved in wheat (*Triticum aestivum*) [10], and grapevine (*Vitis vinifera*) [11]. However, MAX2 plays a role also in the signal transduction of other bioactive molecules, such as karrikins (KAR) [12] or brassinosteroids [13], and rac-GR24 is a mixture of two stereoisomers that can activate responses of both SLs and KAR [14, 15]. Studies using SL biosynthesis mutants in Arabidopsis and rice (*Oryza sativa*) delivered contradictory results about the role of SL in drought response. Arabidopsis SL biosynthesis mutants (*max3* and *4*) were described as possessing increased sensitivity to drought in one report [9], while according to the other study, no differences between WT and SL biosynthesis mutants in drought sensitivity were found (*max1, 3* and *4*) [8]. On the other hand, analysis of rice SL mutants revealed that SL-deficient mutants (*d10* and *d17*) and SL-insensitive mutant (*d3*) were more tolerant to drought than WT [16]. On the other hand, another SL-deficient mutant (*d27*) displayed decreased tolerance to the water-deficit conditions [16], similar to tomato (*Solanum lycopersicum*) and Lotus japonicas, SL-depleted plants exhibited higher susceptibility to drought [17, 18]. It has to be highlighted that biosynthesis mutants cannot be considered fully depleted; thus it was suggested that SL mutants deficient in the signaling components for SLs, such as receptor D14, should be used in studies examining the role of SLs in plants [2]. For example, Arabidopsis mutant *atd14* revealed increased sensitivity to water-deficit conditions due to slower stomatal closure, lower anthocyanin content, faster water losses, and lower photosynthesis efficiency [19, 20]. Similar results were obtained for barley (*Hordeum vulgare*) *hvd14.d* mutant, which exhibited a drought sensitivity phenotype related to lower leaf relative water content (RWC), impaired photosynthesis, disorganization of chloroplast structure, altered stomatal density, and slower closure of stomata in response to drought when compared to the WT. It was also shown that the lower drought resistance of *hvd14.d* plants was due to ABA insensitivity [19]. One explanation of how both phytohormones, SLs and ABA, may contribute to drought response in plants was provided in tomato, where micro(mi)RNA miR156 was suggested to function as mediators between ABA and SLs in controlling stomatal closure [21]. However, SLs may also trigger stomatal closure in an ABA-independent manner [22], so a broader, systemic understanding of SL-dependent drought responses remains to be resolved.

Here, using a combination of transcriptomics and proteomics, we systemically characterize the drought-response(s) of the SL-insensitive mutant *hvd14* relative to WT barley to elucidate the role of SLs under water-deficient conditions. Through these analyses, we resolved a set of transcription factors and defined the systemic molecular changes that significantly enhanced our understanding of the regulatory mechanisms of SL and how barley responds to drought.

## Results

### Mutant *hvd14* is insensitive to SL and more sensitive to drought

**Fig. 1:**
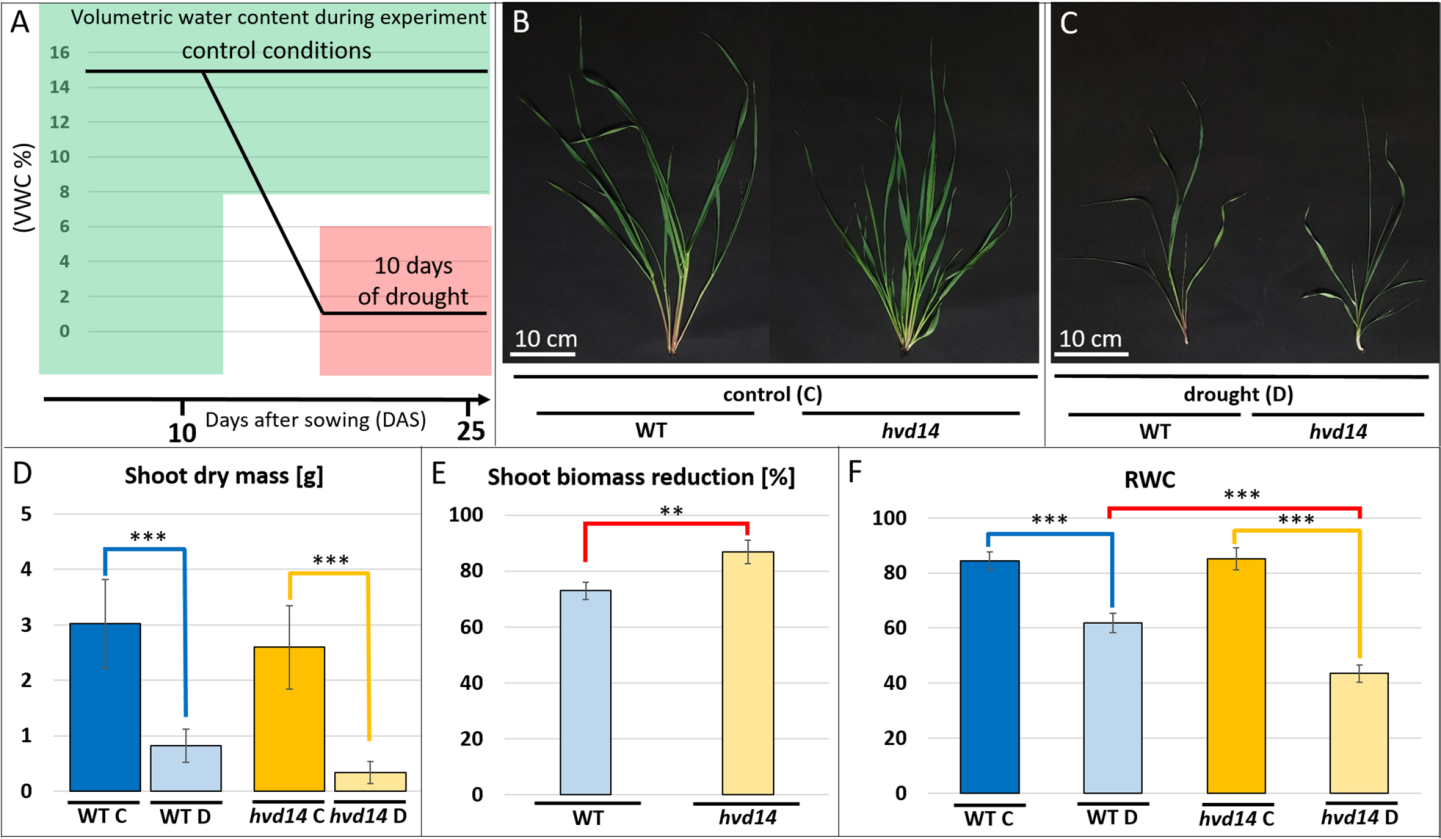
Barley SL insensitive mutant *hvd14* is more sensitive to drought than the WT. **A** Overview of drought stress protocol used: plants were grown under optimal water conditions (15% of volumetric water content, vwc) for ten days after sowing (DAS). Next, plants were divided into two groups: control (**C**) plants that grew for another 15 days (25 DAS) under optimal water conditions and plants exposed to drought (**D**). For stressed plants, the watering was stopped for five days, which allowed to decrease VWC to 1.5–3%, and that value was maintained for another ten days. **B** Control and **C**. stressed plants at the end of the experiment (25 DAS). Effect of drought on **D**. shoot dry mass, **E**. shoot dry mass reduction, and **F**. relative water content (RWC) in leaves of both genotypes. Asterisks indicate statistically significant differences between samples in a paired Student's t-test (*, **, and *** correspond to *p-values* of 0.05 > *p* > 0.01, 0.01 > *p* > 0.001, and *p* < 0.001, respectively)

Mutant *hvd14.d* (referred hereafter as *hvd14*), identified by TILLING, carried the G725A transition, which led to substituting a highly conserved glycine-193 to glutamic acid in SL receptor. This mutation decreased barley sensitivity to synthetic SL analog GR24 in 1 and 10 µM [23]. However, GR24 is a mix of two enantiomers, GR24^5DS^ and GR24^ent−5DS^, that may mimic two phytohormones: SLs and KAR. Therefore, we tested the SL-specific response of *hvd14* to GR24^5DS^ treatment. After 18 days of treatment with 10 µM of GR24^5DS^, a statistically significant reduction in the tillering of wild-type (referred to hereafter as WT) was observed compared to the control treated with 0.01% acetone (1.9 ± 0.44 vs 3 ± 0.41, respectively). At the same time, no differences in tillering were observed between treated and non-treated *hvd14* plants (4 ± 0.45 vs 3.9 ± 0.41) (Supplemental Fig. 1). Thus, decreased SL sensitivity of *hvd14*, which results from the reduced aperture of the HvD14 active site pocket entrance, was confirmed [23]. Further, it was reported that *hvd14* is more sensitive to drought under mild water deficit conditions [19]. To resolve drought stress responses in barley, we applied drought stress to barley seedlings (10 days after sowing, DAS) for 15 days (Fig. 1A). This resulted in noticeable developmental differences observed between control (25 DAS; C) and stressed plants (25 DAS; drought; D) due to inhibited growth of plants exposed to drought (Fig. 1B, C). Drought caused more substantial biomass reduction and lower relative water content (RWC) in *hvd14* leaves than WT (Fig. 1D**-**F). We also performed an additional water-withholding experiment to compare the survival rate of WT and *hvd14.* This found a higher drought sensitivity in *hvd14* (survival rate 24.1% ± 7.07) when compared to WT (survival rate 82.5% ± 9.38) (Supplemental Fig. 2).

**Fig. 2:**
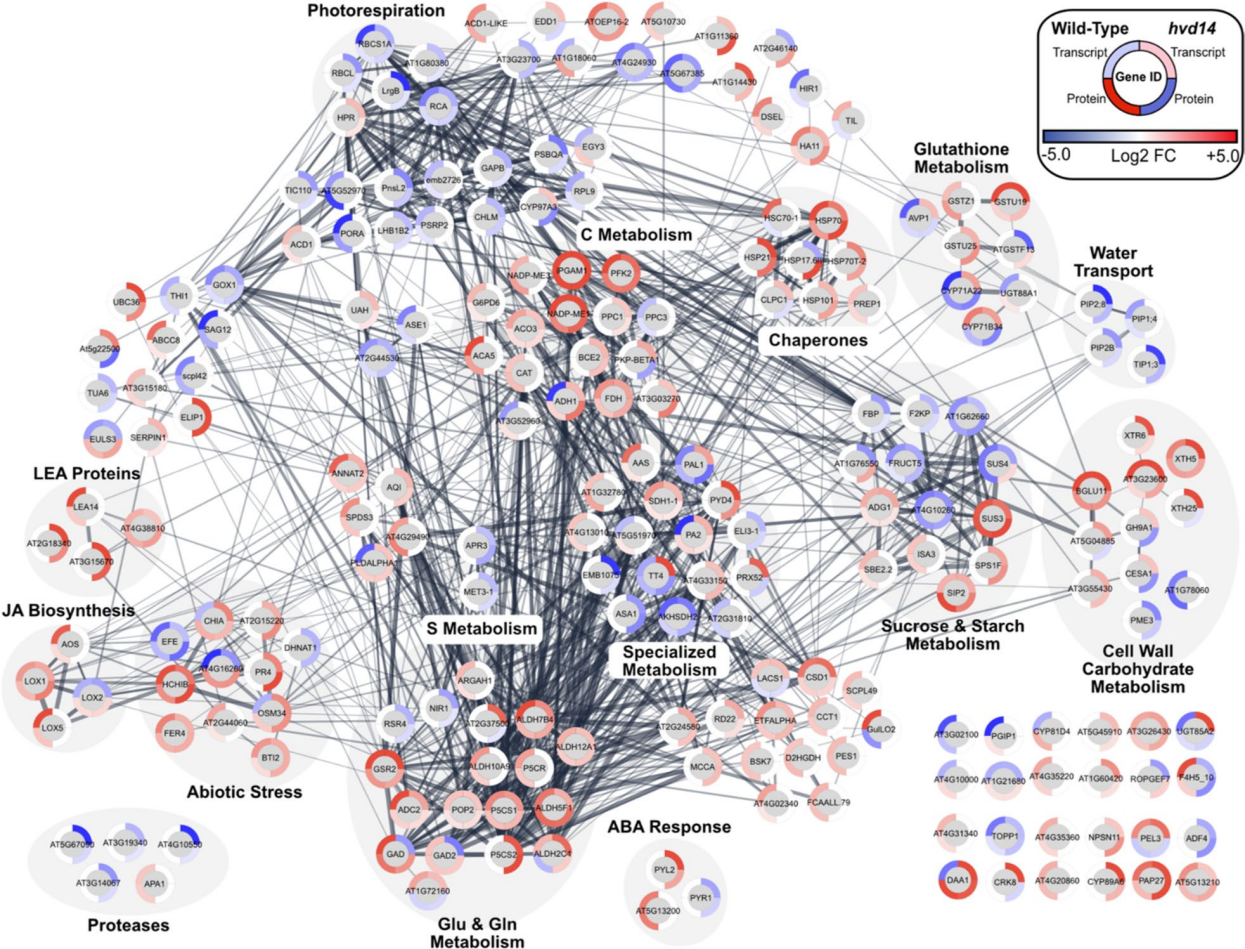
Association network of drought-responsive genes in wild-type and *hvd14*. Arabidopsis homologs for all barley genes exhibiting a significant change in transcript- and protein abundance are depicted (Supplemental Data 3). The association network was constructed in Cytoscape (v. 3.9) using a combination of the string-db and enhancedGraphics plug-ins and a stringdb edge score of > 0.4. Color scale depicts log_2_ FC in abundance

### Analysis of SL-dependent transcriptome response to drought

Transcriptome analyses were performed to uncover the molecular basis of hvd14 drought sensitivity. First, the comparison of WT plants grown under control and stressed conditions revealed 5689 WT drought-responsive (DR) differentially expressed genes (DEG); 2670 up-, and 3019 down-regulated). In *hvd14* leaves, 4096 DEG (1699 up-, and 2397 down-regulated *hvd14_*DR) in response to drought were found (Supplemental Fig. 3, Supplemental Data 1). We considered DEG when log2FC (fold change) ≥ 1 (up-regulated genes) or log2(FC) ≤ -1 (down-regulated genes) and adjusted *p-value* ≤ 0.01. Both genotypes shared less than 50% DR genes: 1216 common up-regulated genes (1400 WT specific; 449 *hvd14* specific) and 1768 down-regulated genes (1210 WT specific; 602 *hvd14* specific) (Supplemental Fig. 3, Supplemental Data 1).

**Fig. 3:**
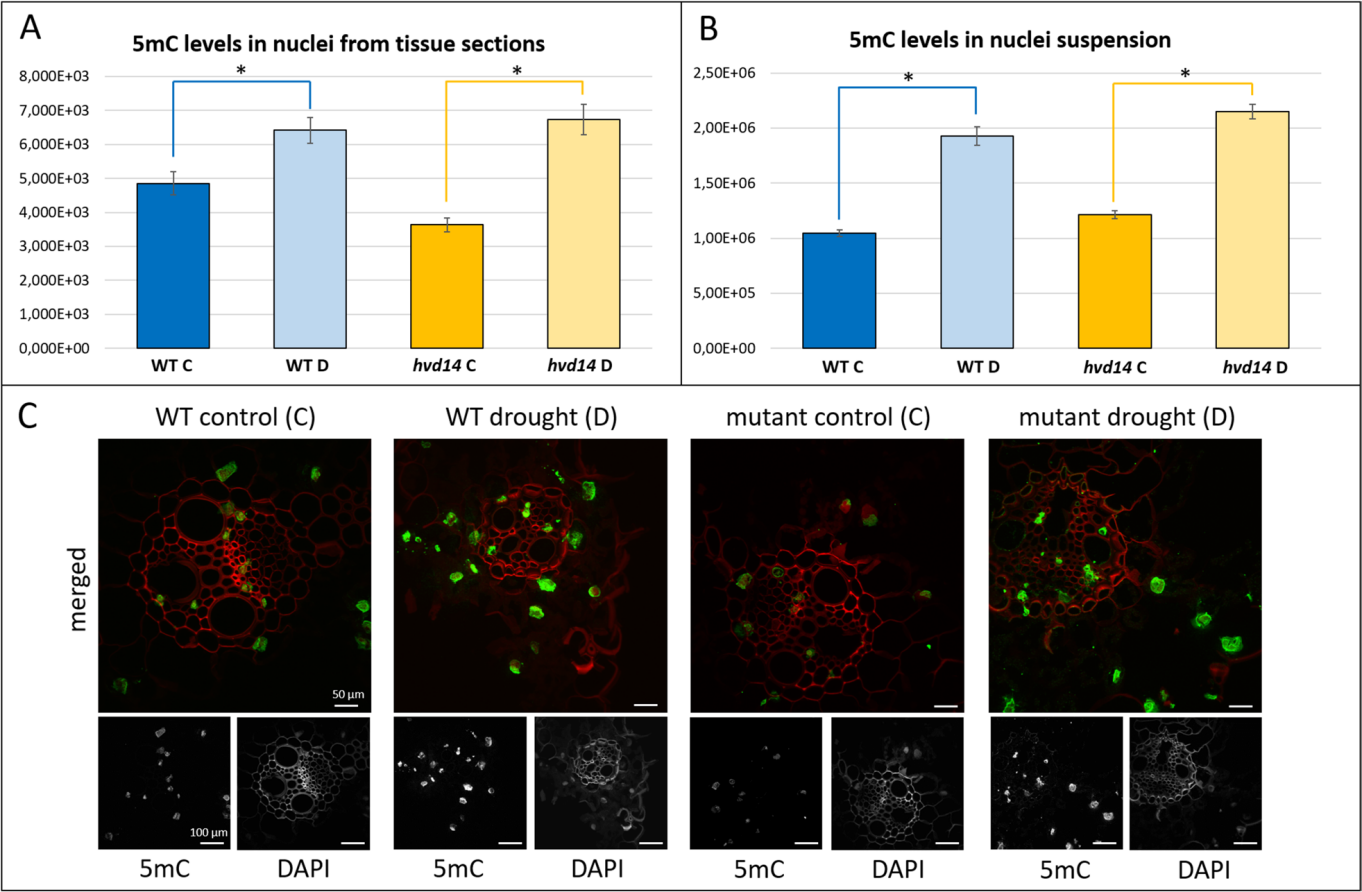
The 5-methylcytosine (5mC) level in leaf tissue sections and leaf nuclei suspensions. **A** Analysis of 5mC levels in nuclei from leaves indicates the increase of 5mC level in stressed plants of both genotypes compared to control plants when measured from tissue sections using CLSM and **B** from nuclei suspension using HCSFM. **C** Cross-sections of leaves of both genotypes under control and stress conditions. The green color indicates the Alexa 488 fluorescence (5mC), and the red color refers to DAPI fluorescence (false color) and cell walls autofluorescence. Asterisks indicate statistically significant differences between samples in a paired Student's t-test (* correspond to *p-value* of *p* < 0.05)

### Analysis of SL-dependent proteome response to drought

The same strategy applied for transcriptome analyses was used to identify the differentially abundant proteins (DAP) related to the drought response of barley plants. Here, we utilized a log2FC ≥ 0.58 (corresponding to a 1.5-fold change) ≤ log2FC -0.58, and *q-value* ≤ 0.05 DAP selection threshold. Drought-stressed WT plants revealed 440 (288 up- and 152 down-regulated) DAP, whereas, in the case of the *hvd14*, 651 DAPs (315 up- and 336 down-regulated) were found in response to drought (*hvd14* DR). Comparing WT DR vs *hvd14* DR revealed a set of proteins involved in barley drought response that differed among both genotypes, named drought response specific (DRS). This included: 132 (95 up-, 37 down-regulated) and 274 (109 up-, 165 down-regulated) DAPs for WT and *hvd14*, respectively. On the other hand, 175 up- and 90 down-regulated DAPs were common for both genotypes in response to drought (Supplemental Fig. 3, Supplemental Data 2).

### Transcriptome–proteome intersections reveal core drought response changes

To distil out how drought impacts barley across multiple levels of cellular regulation, we next examined the drought-response landscape of WT and *hvd14* plants for genes that demonstrate significant changes in both the transcriptome (Log_2_FC > 1.5; *p-value* < 0.01) and proteome (Log_2_FC > 0.58; *q-value* < 0.05). Here, we found a total of 135 and 151 genes in WT and *hvd14*, respectively, which are differentially regulated in response to drought conditions (Supplemental Data 3). To contextualize these changes and resolve genes that exhibit transcript-protein regulation, we next generated a STRING-DB association network using Arabidopsis homologs (Fig. 2; Supplemental Data 3). This resolved a number of gene clusters involved in carbon (C), sulfur (S), specialized, starch and sucrose, cell wall carbohydrate and glutathione metabolism along with clusters of ABA and jasmonic acid (JA) responsive genes, which align with our phytohormone profiling of WT and *hvd14* plants (described below). Our association network analysis also resolved clusters of proteases, water transport and fructose metabolism genes that are specifically down-regulated and cell wall carbohydrate metabolism and chaperone gene clusters that are specifically up-regulated in the *hvd14* plants under drought stress, indicating that loss of SL-signaling directly impacts multiple core adaptive drought responses, and does so at both the transcriptome and proteome-level (Fig. 2).

### Cytosine methylation induced by drought is non-SL dependent

Our transcriptomic data found the total number of DEGs and the number of genotype-specific DEGs to be higher in WT compared to *hvd14* (5689 vs 4096 and 2610 vs 1051, respectively), indicating a weaker transcriptomic response in *hvd14*. It has been shown that the methylation of DNA plays a crucial role in the modulation of gene expression in barley response to drought [24]. DNA methylation occurs via the addition of a methyl group (-CH_3_) at the 5′ position of cytosine, resulting in 5-methylcytosine (5mC) [25]. We then measured the 5mC levels in nuclei from tissue sections using confocal laser scanning microscopy (CLSM) and from nuclei suspension using high content screening fluorescence microscopy (HCSFM) equipped with automated autofocus. Results obtained with both methods were consistent and indicated that drought significantly elevated the level of DNA methylation in leaves of both genotypes (Fig. 3). In WT plants, 5mC levels increased by 56% or 83%, respectively, according to the CLSM and HCSFM methods. Alternatively, *hvd14* plants were found to respond to drought by enhancing DNA methylation by 68% or 78%, respectively (Fig. 3). Thus, drought stress causes a similar increase of DNA methylation independent of genotype, suggesting the lower number of DRS DEGs in the *hvd14* may not be exclusively due to a disturbance in DNA methylation mechanisms. The lack of DNA methylation differences does not exclude the possibility that different genome regions are methylated in both genotypes, thus affecting the expression of specific genes; however, this is outside the scope of utilized methods here.

### SL-dependent TFs involved in barley response to drought

With transcription factors (TFs) playing a crucial role in coordinating global plant responses to diverse stimuli we next queried our data for quantitative change in TFs. Among 2610 DRS WT genes (adjusted *p-value* ≤ 0.01; Log2FC > 1.0 or < -1.0), there were 134 (95 up- and 39 down-regulated) TFs. Of these, many were involved in: the abscisic acid (ABA) signaling pathway and response to drought (39), response to other abiotic and biotic stresses (10), different developmental processes (10), and reactions to various phytohormones other than ABA (10), with a total of 26 of the identified TFs having no annotated biological function (Supplemental Data 4).

Next, we examined the promoter regions (1500 bp) of the genes corresponding to all significantly changing transcripts and proteins from WT plants to identify putative TF binding sites that may align with our differentially expressed TFs (Supplemental Data 5). Binding sites for 27 identified TFs (21 up- and six down-regulated) were found among DEG and genes encoding DAP, which were changed explicitly by drought in WT plants (Table 1). Most of the TFs up-regulated in WT plants in response to drought is related to the ABA signaling (Table 1). Gene ontology (GO) enrichment analysis (FDR ≤ 0.05) of TF targets revealed their roles are related to response to reactive oxygen species, response to ABA, JA biosynthetic process, and response to water deprivation (Supplemental Data 6). Collectively, this suggests that *hvd14* has a lower sensitivity to ABA and a lack of ABA-dependent TF activation, leading to the lower drought resistance of *hvd14*. Whereas in WT plants, identified ABA-dependent TFs likely regulate targets involved in phytohormone biosynthesis, response to oxidative species, cell wall remodeling and lipid metabolism, which play a role in wax deposition on the leaf surface, contributing to enhanced drought tolerance relative to *hvd14*.

**Table 1:** WT DRS TFs and the number of their targets which are specifically regulated only in WT when compared to the mutant, in response to drought

	**HORVU id**	**WT_D vs WT_C**		**No. of DRS WT genes**	**No. of DRS WT proteins**	**Arabidopsis homologue**	**Description (PlantTFDB / uniprot)**
		**log2FC**	**adj.pval**				
up-regulated	HORVU4Hr1G023110 (MLOC_58641)	1,19	0,00	787	59	AT4G27950 CRF4	Component of the cytokinin signaling pathway involved in cotyledons, leaves, and embryos development
	HORVU1Hr1G060490 (MLOC_77405)	1,00	0,00	733	47	AT2G40340 ERF48/ DREB2C	Binds to the C-repeat/DRE element mediates high salinity- and abscisic acid-inducible transcription
	HORVU0Hr1G007050 (MLOC_24530)	3,52	0,00	477	41	AT3G15210 ERF4	Involved in response to abscisic acid
	HORVU1Hr1G090030 (MLOC_6711)	2,45	0,00	122	6	AT2G46270 GBF3	Encodes a bZIP G-box binding protein whose expression is induced by ABA
	HORVU5Hr1G095630 (MLOC_61901)	1,19	0,00	119	10	AT1G69120 AP1/AGL7	Promotes early floral meristem identity in synergy with LEAFY
	HORVU1Hr1G049840 (MLOC_58950)	4,16	0,00	112	7	AT4G17980 ANAC071	Required for the auxin- mediated promotion of vascular tissue proliferation during hypocotyl graft attachment
	HORVU6Hr1G028790 (MLOC_60890)	1,63	0,00	100	7	AT1G80840 WRKY40	Response to ABA; response to water deprivation
	HORVU5Hr1G070800 (MLOC_62335)	1,42	0,00	87	9	AT1G69010 BIM2	Positive brassinosteroid-signaling protein
	HORVU7Hr1G026940 (MLOC_81350)	1,08	0,00	85	11	AT2G23340 DEAR3	Involved in ethylene-activated signaling pathway
	HORVU2Hr1G021080 (MLOC_51930)	1,02	0,00	84	7	AT3G62420 BZIP53	Transcription activator that binds ABA-responsive elements (ABREs)
	HORVU6Hr1G068100 (MLOC_14844)	1,09	0,00	78	5	AT5G45580 GFR	DNA-binding transcription factor activity
	HORVU5Hr1G097500 (MLOC_65033)	1,80	0,00	71	7	AT1G27730 STZ/ZAT10	Response to ABA; response to water deprivation; response to oxidative stress
	HORVU1Hr1G065570 (MLOC_36942)	1,41	0,00	68	5	AT5G64060 NAC103	DNA-binding transcription factor activity
	HORVU0Hr1G001230 (MLOC_6041)	1,39	0,00	62	5	AT5G06100 MYB33	Positive regulation of ABA-activated signaling pathway
	HORVU2Hr1G017400 (MLOC_65400)	2,14	0,00	58	7	AT5G39610 NAC2/ORE1	Accumulates in response to ABA
	HORVU6Hr1G065430 (MLOC_62554)	5,02	0,00	57	0	AT5G52020	Involved in the regulation of gene expression by stress factors and by components of stress signal transduction pathways
	HORVU3Hr1G033740 (MLOC_68299)	1,74	0,00	55	6	AT1G62300 WRKY6	TF involved in the control of processes related to senescence and pathogen defense
	HORVU3Hr1G014140 (MLOC_65101)	4,06	0,00	44	2	AT1G61110 NAC25	Transcription activator of the abscisic aldehyde oxidase AAO3 (leads to increased levels of the ABA)
	HORVU5Hr1G049880 (MLOC_64817)	1,14	0,00	43	0	AT4G37260 MYB73	Involved in response to abscisic acid
	HORVU4Hr1G052330 (MLOC_64008)	1,08	0,00	36	0	AT5G06950 TGA2	Binds to the as-1-like cis elements mediate auxin- and salicylic acid-inducible transcription
	HORVU6Hr1G035470 (MLOC_7426)	2,27	0,00	23	0	AT5G67300 MYB44	Represses the expression of protein phosphatases 2C in response to abscisic acid
down-regulated	HORVU7Hr1G012840 (MLOC_15776)	-1,58	0,00	524	39	AT5G42520 BPC6	Specifically binds to GA-rich elements (GAGA-repeats) present in regulatory sequences of genes involved in developmental processes
	HORVU7Hr1G096430 (MLOC_54829)	-1,37	0,00	167	8	AT3G13040	Involved in phosphate starvation signaling
	HORVU2Hr1G113940 (MLOC_81350)	-1,03	0,00	85	11	AT5G67190 DEAR2	Involved in ethylene-activated signaling pathway
	HORVU6Hr1G092750 (MLOC_29808)	-2,04	0,00	58	4	AT5G04390	Transcription regulatory region sequence-specific DNA binding
	HORVU1Hr1G076690 (MLOC_38232)	-2,06	0,00	45	0	AT2G33860 ARF3	Auxin response factors (ARFs) are transcriptional factors that bind specifically to the DNA sequence 5'-TGTCTC-3' found in the auxin-responsive promoter elements (AuxREs). Could act as transcriptional activator or repressor
	HORVU4Hr1G050190 (MLOC_60045)	-1,56	0,00	44	3	AT1G06850 BZIP52	Involved in vascular development and shoot tissue organization

Underlined Arabidopsis genes were described as TFs which are involved in SL-dependent response to drought in Arabidopsis [45]

### Multiple phytohormones affected by drought in an SL-dependent manner

**Fig. 4:**
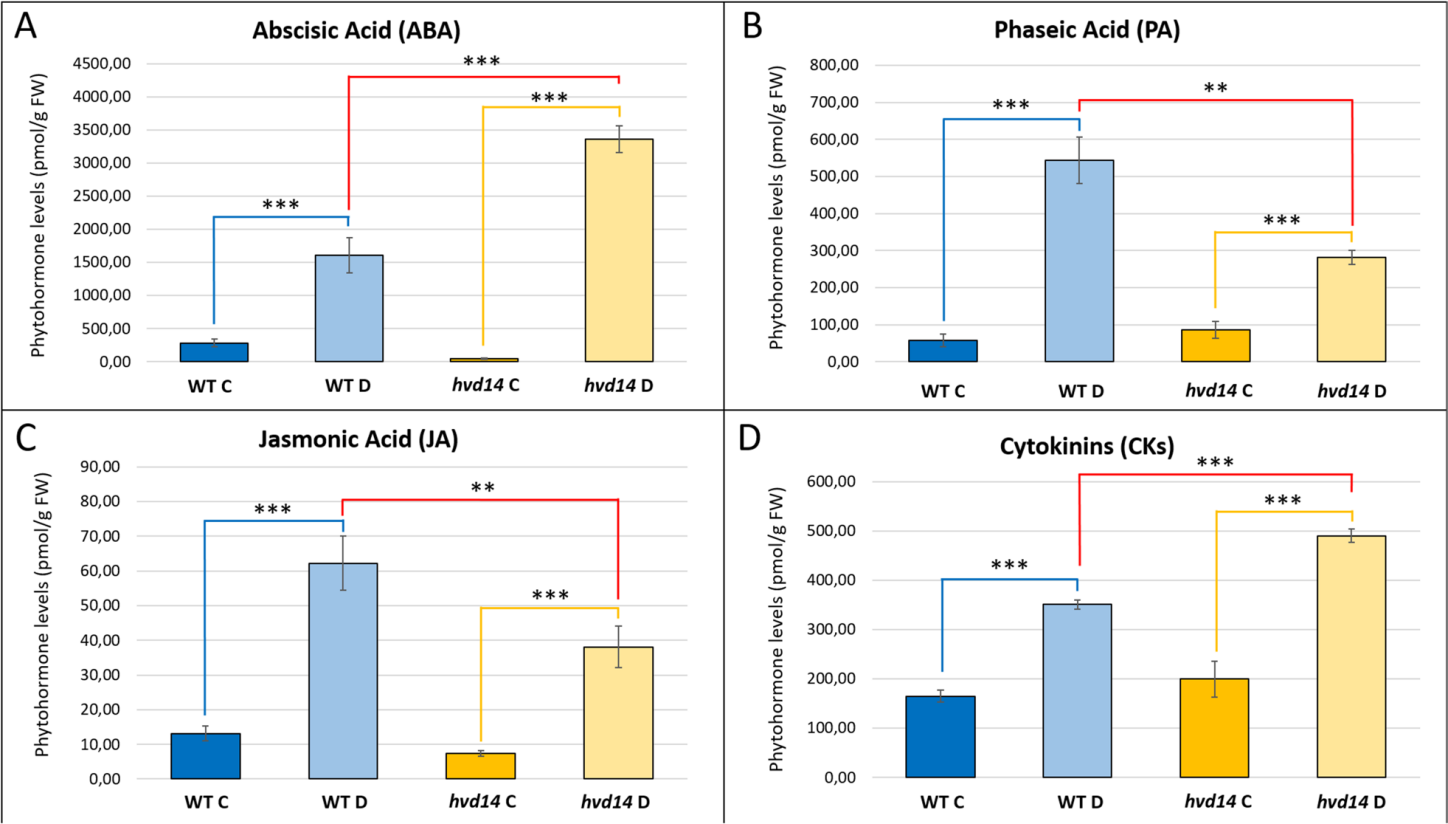
Phytohormone profile of WT and *hvd14* under control and drought conditions. **A** Level of abscisic acid (ABA) in the shoot of both genotypes in control (**C**) and stressed (**D**) plants. **B** Drought increased the phaseic acid (PA) level in both genotypes. **C** Weaker response to the drought of the *hvd14* was observed in the case of jasmonic acid (JA) accumulation. **D**. Drought increases the cytokinins (CKs) level. Asterisks indicate statistically significant differences between samples in a paired Student's t-test (*, **, and *** correspond to *p-values* of 0.05 > *p* > 0.01, 0.01 > *p* > 0.001, and *p* < 0.001, respectively)

Given a large number of phytohormone-related genes exhibiting transcriptomic and/or proteomic changes in response to drought, we next assessed the phytohormone profiles of control and stressed plants of both genotypes. Here, we find a similar pattern of ABA fluctuation in response to drought in WT and *hvd14*. In both genotypes, ABA content increased significantly compared to control plants; however, more pronounced changes were observed in *hvd14* (69 FC) than in WT (5.6 FC), resulting in a twofold higher ABA content (3362.00 vs 1603.55 pmol/g FW) in *hvd14* shoots under drought stress (Fig. 4A). The differences in ABA content between both genotypes were detected under control conditions. The content of phaseic acid (PA), a bioactive ABA metabolite [26], also increased in response to drought. However, PA content after the drought was almost two times higher in WT (543.47 pmol/g FW) than in the *hvd14* at the same time (281.57 pmol/g FW) (Fig. 4B). These results indicated that *hvd14* has a lower ABA sensitivity, like that previously reported for other SL mutants in Arabidopsis [20] and barley [19]. We also observe an overall decrease in the ABA-response related transcriptional response of *hvd14*. Under drought conditions, 134 genes (of 1400; 6.7%) induced only in WT were assigned into GO categories such as 'abscisic acid' and 'abscisic acid-activated signaling pathway'. On the other hand, only 16 (of 449; 3.5%) genes with those GO annotations were up-regulated during the drought response of *hvd14* (Supplemental Fig. 4A, Supplemental Data 1).

Further, drought stress significantly elevated JA content in the shoot tissue of both genotypes (Fig. 4C). JA level achieved greater concentration in WT (62.25 pmol/g FW) than *hvd14* (38.11 pmol/g FW). These differences in JA content were also reflected in observed transcriptome changes, with the up-regulated expression of 33 genes (of 1400; 2.35%) annotated to 'response to jasmonic acid', 'jasmonic acid biosynthetic process, and 'jasmonic acid metabolic process' GO terms found in the DRS WT list, whereas only 10 (of 449; 0.42%) were present in up-regulated DRS *hvd14* (Supplemental Fig. 4B, Supplemental Data 1). We also find four lipoxygenase proteins, which may be involved in JA biosynthesis up-regulated by drought in WT only, along with the barley homolog of *OXOPHYTODIENOATE-REDUCTASE 3* (*OPR3*), which encodes a 12-oxophytodienoate reductase (HORVU7Hr1G095960) that is required for jasmonate biosynthesis [27] (Supplemental Fig. 4C). Moreover, it was shown that both JA and its precursor, oxylipin 12-oxo-phytodienoic acid (OPDA), play a role in stomata closure [28–30].

Both WT and *hvd14* genotypes significantly increased the cytokinin (CKs) content in response to drought. However, we detected a significantly higher CK level in the *hvd14* shoot than WT (490.16 and 350.66 pmol/g FW, respectively) (Fig. 4D). Those differences can be linked with the up-regulated expression of HORVU3Hr1G066810 encoding cytokinin riboside 5'-monophosphate phosphoribohydrolase in the *hvd14*. This enzyme converts the inactive CK nucleotides to biologically active free-base forms [31]. On the other hand, the expression of a gene encoding another enzyme involved in CK activation (HORVU1Hr1G041640), was down-regulated only in WT in response to drought (Supplemental Fig. 4D).

### Drought stress reveals differential wax deposition between WT and *hvd14*

One of the main drought-survival strategies is reducing water losses, which can be achieved by increased wax deposition and changing the wax composition on the cell surface. Activation of wax biosynthesis pathways and mechanisms of wax transport to the cell wall was described as ABA-dependent and induced by drought in barley [32]. The thinner cuticle layer on the abaxial epidermal cell in *hvd14* compared to WT was previously observed in plants exposed to drought [19]. Further analysis of the genes described as crucial for wax biosynthesis [32] or annotated to the 'cutin biosynthetic process' and 'cuticle development' revealed a group of eight genes up-regulated by drought in WT, but not in *hvd14* (Supplemental Data 7).

**Fig. 5:**
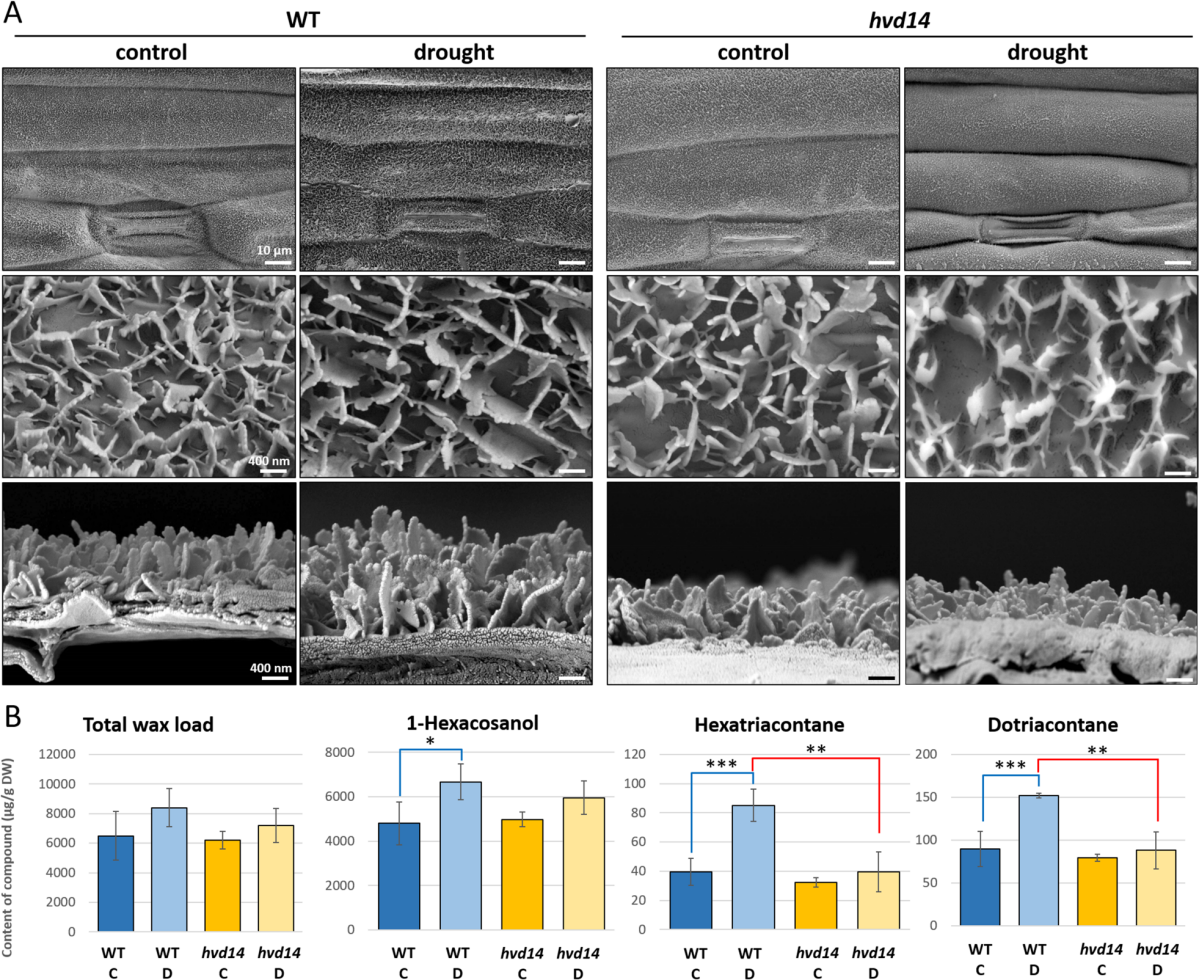
Drought affects wax biosynthesis in barley. **A** Differences in the thickness and structure of wax layer on the leaf surface of WT and *hvd14* under control and drought conditions. In WT, the thickness and number of wax crystals increased after exposition to drought, which was not the case in the *hvd14*. **B** Drought increased the content of 1-hexaconasol, hexatriacontane and dotriacontane in the epicuticular wax layer in WT. Asterisks indicate statistically significant differences between samples in a paired Student’s t-test (*, **, and *** correspond to *p-values* of 0.05 > *p* > 0.01, 0.01 > *p* > 0.001, and *p* < 0.001, respectively)

Analysis of the epicuticular wax layer using a scanning electron microscope (SEM) confirmed the increased deposition of wax in WT exposed to drought. That high deposition was visible in the wax layer's thickening and the density of wax crystals at the abaxial leaf surface (Fig. 5A). The wax layer at the abaxial surface of the control and stressed *hvd14* leaves was similar (Fig. 5A), which aligns with our transcriptomic findings. Metabolite analysis found no statistically significant differences in the chemical composition of wax deposition between control and stressed plants in either genotype except for an increase in the content of 1-hexacosanol, the main component of the barley wax layer [33]. Similar results were obtained for two other barley wax components, hexatriacontane and dotriacontane, in which abundance was induced by drought in WT stronger, compared to *hvd14* (Fig. 5B, Supplemental Data 7B).

### SL-deficient *hvd14* exhibits reduced ROS scavenging

**Fig. 6:**
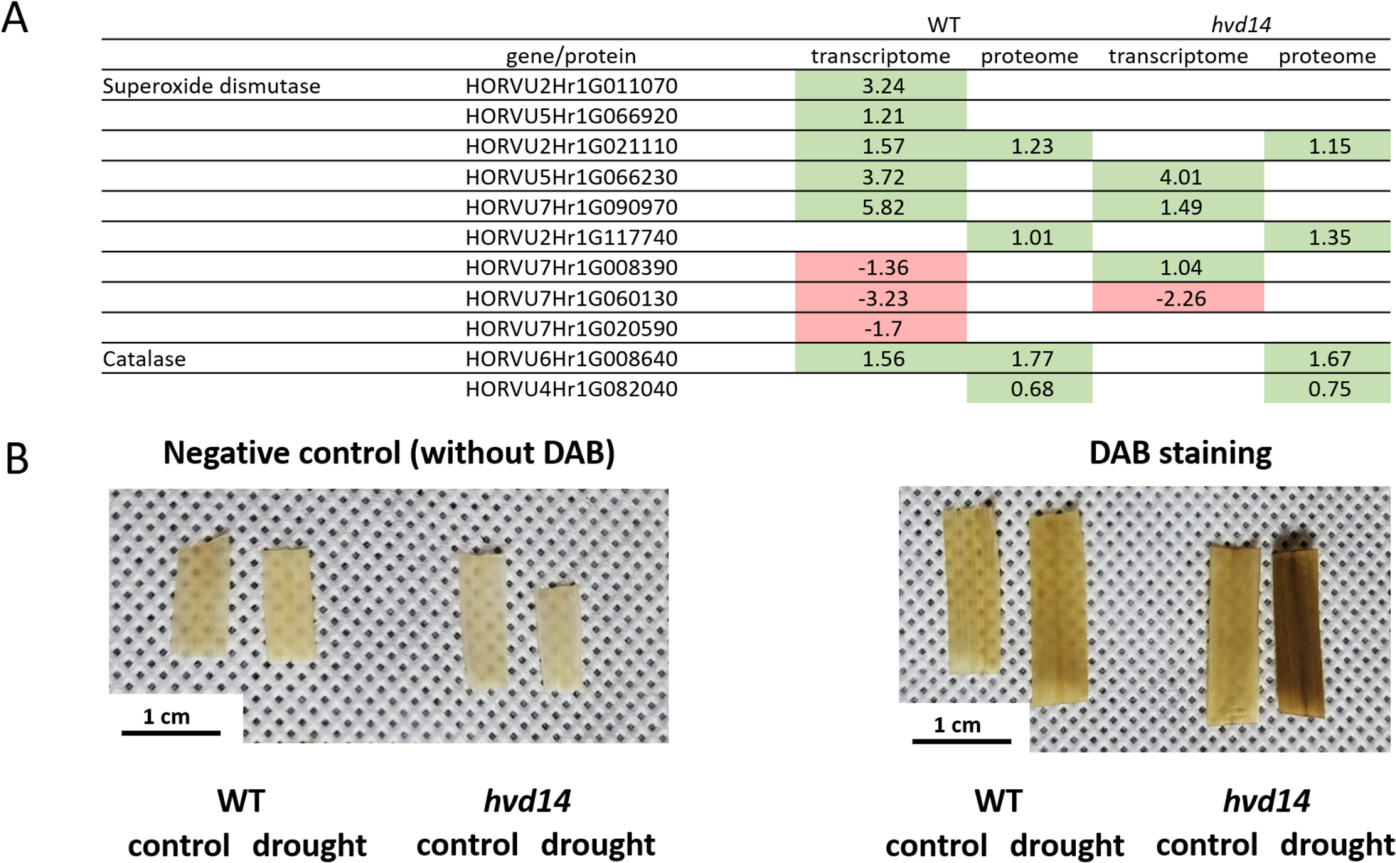
Reactive oxygen species (ROS) scavenging mechanisms are disturbed in the *hvd14* mutant. **A** Genes and proteins involved in controlling ROS level in barley are activated in WT but not in *hvd14* in response to drought. **B** After exposition to the drought, the 3,3'-diaminobenzidine (DAB) staining revealed higher hydrogen peroxide content in mutant leaves than in WT leaves

Reactive oxygen species (ROS), such as superoxide anion, hydroxyl radical, or hydrogen peroxide, act as alarm signal which activates the plant defence strategies. However, increased ROS levels are toxic for cells, and plants develop various ROS scavenging mechanisms, including superoxide dismutase and catalase [34]. Representatives from both groups of enzymes were upregulated by drought in barley. Two superoxide dismutases (HORVU2Hr1G011070, HORVU5Hr1G066920) were induced explicitly by drought exclusively in WT. In contrast, the expression of four superoxide dismutases was induced by drought in both genotypes (HORVU2Hr1G021110; HORVU5Hr1G066230; HORVU7Hr1G090970; HORVU2Hr1G117740) (Fig. 6A). These results indicate that *hvd14* cannot activate mechanisms of ROS scavenging with the same efficiency as WT, which was confirmed using 3,3'-diaminobenzidine (DAB) staining against hydrogen peroxide [35]. The staining of WT and *hvd14* leaves of control and stressed plants indicate the accumulation of hydrogen peroxide caused by drought in both genotypes. However, a higher level of hydrogen peroxide was observed in *hvd14* leaves compared to WT (Fig. 6B).

## Discussion

### Drought-sensitivity of *hvd14* may be due to lower sensitivity to ABA

ABA plays a crucial role in plant response to water-limited conditions by activating drought-responsive genes and controlling physiological processes, including stomatal closure [36]. The increased drought susceptibility of SL mutants was previously linked to lower ABA sensitivity, which resulted in the weaker expression of ABA-induced genes in the Arabidopsis *atd14* mutant than in the WT [20]. Similarly, the barley mutant *hvd14* was also found to be less ABA-sensitive than the WT during germination [19]. Both *d14* mutants are linked with the impaired stomatal closure mediated by ABA [19, 20]. Thus, it can be predicted that the drought-sensitive phenotype of SL-insensitive mutants is related to the faster water loss (due to slower stomata closure and thinner wax laver), which damages the photosynthetic apparatus and results in growth inhibition and finally death.

Our molecular findings strengthen the hypothesis that *hvd14* possesses reduced ABA sensitivity, given that after drought, ABA levels increased in the shoot of *hvd14* tissue threefold compared to WT under limited water conditions. Interestingly, the expression of two main enzymes involved in ABA biosynthesis (ABA1: HORVU2Hr1G078840, AT5G67030 and ABA2: HORVU3Hr1G046320, AT1G52340) were down-regulated in *hvd14* after exposition to the drought indicating that the positive feedback mechanism regulating ABA biosynthesis remains active in *hvd14.* At the same time, we find levels of phaseic acid (PA), an ABA metabolite, to be twofold lower in *hvd14* relative to WT (Fig. 4). Thus, higher ABA concentrations in *hvd14* after a drought may result from impaired ABA catabolism. Indeed, drought more readily represses the expression of abscisic acid 8'-hydroxylase (HvABA8'OH-2; HORVU5Hr1G068330; AT3G19270), one of the ABA catabolic enzymes, in *hvd14* compared to the WT (-2.55 vs -1.83 log_2_FC, respectively). Similar results were obtained for Arabidopsis, where ABA catabolic enzymes were down-regulated in drought-treated *atd14*, not WT plants [20]. Although ABA content is elevated in *hvd14* in response to drought, our results showed that ABA is catabolized much less effectively into PA, which in Arabidopsis, results in higher drought sensitivity [37]. Moreover, the application of GR24 induced the expression of ABA catabolism in the grapevine (*Vitis vinifera* L.) [38]. Thus, the drought-sensitive phenotype of *hvd14* may arise in lower sensitivity to both ABA and PA.

At the molecular level, we observe parallel transcriptome and proteome changes in key ABA signaling pathway proteins (PYR1; AT2G26040) and (PYL2; AT4G17870) are both known receptors of ABA. Interestingly, we see an in-parallel transcriptome and proteome down- and up-regulation of the ABA receptor PYR1 and PYL2, suggesting that SL may specifically change how ABA is perceived at the molecular level. Transcriptomic analyses in Arabidopsis indicated that the SL repressor (SMXL6) could regulate the expression of another ABA receptor (PYL9) [7]. Interestingly, the design and implementation of ABA agonists have found that molecular design can differentially impact how receptors can be stimulated [39]. The down-regulation of the main ABA receptor PYR1 likely drives the phenotypic responses that we are seeing in conjunction with lower ABA levels; however, the up-regulation of PYL2, a PYR1-related receptor, remains intriguing. That disrupted ABA perception in *hvd14* is reflected in the drought-induced up-regulated expression of protein phosphatases 2C (HORVU1Hr1G080290 and HORVU3Hr1G067380) homologs of AtHAB1 (HYPERSENSITIVE TO ABA1, AT1G72770) and HORVU3Hr1G050340 homolog of AtHAB2 (AT1G17550), the negative regulators of ABA signaling [40, 41], observed only in WT.

### Molecular underpinnings of SL-dependent drought response in barley

Our comprehensive systems-level study, combining transcriptomic and proteomic analyses, has revealed a diverse array of drought responses that are dependent on SL signaling. We find a decrease in ABA sensitivity reflected in the lower induction of ABA-dependent genes and proteins in the *hvd14* in response to drought, including TFs. Identifying genes and proteins that are activated/deactivated in response to drought, specifically in WT but not in the SL-insensitive mutant, allows us to propose a mechanism of SL-dependent barley drought response. Our finding that DNA methylation remains invariable in response to drought between *hvd14* and WT suggests that increased drought sensitivity of *hvd14* may result from impaired response activation rather than the general disturbance of transcriptional and post-translational regulatory mechanisms.

### Transcription factors

Our results identified 27 SL-dependent TFs involved in barley response to drought (Table 1). In silico promoter analysis finds that these 27 TFs can potentially bind the promoter region of 67% WT DRS genes (1752 from 2610) and 77% WT DRS proteins (133 from 172), indicating their significant role in regulating barley response to drought. Moreover, some of the identified WT DRS TFs (HORVU2Hr1G021080, HORVU6Hr1G028790; HORVU7Hr1G096430) were previously reported as involved in barley drought response [42–44]. Their Arabidopsis homologs (AT3G62420, AT1G80840, AT3G13040, respectively) have also been characterized as involved in ABA and/or drought response (Table 1). Still, what is more important, seven of them (underlined in Table 1) were recently identified as involved in D14-dependent response to drought in Arabidopsis [45], which provides solid experimental confirmation of our in silico predictions. Interestingly, one of that homologs – BIM2 (AT1G69010/HORVU5Hr1G070800) interacts with BES1, a target of SL signalling components, and plays an important role in D14-dependent transcriptional responses [45]. Other identified TFs were not previously linked to barley drought resistance or SL signaling. However, for the Arabidopsis homologs of those TFs, some suggestions that they may be involved in plant adaptation to drought stress were postulated (Table 1). Our independent findings that they are involved in drought responses in barley reinforce this proposed function. At the same time, our use of a *hvd14* mutant further connects these TFs with SLs. However, further functional analyses of identified TFs are required to confirm their role in SL-dependent barley response to drought, which is beyond the scope of this study.

Interestingly, many of the identified WT DRS TFs (12 of 27) seem to be ABA-responsive (Table 1), further connecting our ABA measurements to the decreased ABA sensitivity in *hvd14* plants. For example, the HORVU6Hr1G028790 is the homolog of WRKY40 (AT1G80840), a well-known transcriptional regulator of ABA signal transduction in Arabidopsis [46]. This further suggests that lower ABA sensitivity in *hvd14* creates a higher drought susceptibility and implicates several exciting candidates for future characterization. Moreover, the results indicate that the drought experiments for *d14* mutants may be the experimental setups that enable further characterization of ABA and SLs crosstalk.

### Wax biosynthesis

Our finding of disrupted wax biosynthesis and deposition in the *hvd14* leaves exposed to drought was previously described [19]. However, here GC–MS metabolomics analyses revealed the higher content of three components of the barley wax layer (1-hexacosanol, hexatriacontane, and dotriacontane) in drought-stressed WT plants when compared to *hvd14*, which agrees with our transcriptomic data. Analysis of the genes crucial to wax biosynthesis [32] or annotated to 'cutin biosynthetic processes' and 'cuticle development' revealed a group of eight genes up-regulated by drought stress in WT, but not in *hvd14*. Among them, an Arabidopsis *ECERIFERUM* (*CER;* AT1G02205) homolog HORVU6Hr1G089980, which is involved in the wax biosynthesis pathway [47], revealed high drought-induced expression (log_2_FC = 7.8) in WT only. On the other hand, a set of 11 genes involved in wax biosynthesis, including other homologs of Arabidopsis *CER* (HORVU1Hr1G039830), were induced by drought in both genotypes (Supplemental Data 7). These results indicate that *hvd14* can also increase wax biosynthesis in response to drought, but to a lesser extent than WT. Moreover, two independent mechanisms regulating wax biosynthesis and deposition induced by drought are present in barley, and one of them depends on SLs.

### ROS-Scavenging and glutathione metabolism

A comparison between WT and *hvd14* under drought stress conditions indicates that the higher *hvd14* drought sensitivity also relates to less efficient ROS scavenging. We find a drought-induced accumulation of hydrogen peroxide (H_2_O_2_) observed in *hvd14* leaf tissue, which is higher than the WT. It is well known that SL treatment may improve plant response to various stresses by decreasing H_2_O_2_ and superoxide anion content [48]. Similarly, in barley seedlings subjected to cadmium stress, elevated hydrogen peroxide was observed, which was decreased when exogenous GR24 was applied [49]. On the other hand, SLs require H_2_O_2_ synthesis to drive stomatal closure in an ABA-independent manner [22]. Thus, our data suggest that SLs may influence ROS content in various physiological processes through H_2_O_2_, which is supported by observed abundance changes in superoxide dismutases and catalases (Fig. 6A).

Another molecule that acts as an antioxidant is glutathione, which was also proposed to be involved in ABA signal transduction [50]. One of the glutathione S-transferase (HORVU2Hr1G124300; AT1G78380; GSTU19) is known to be involved in plant response to drought, and was highly induced by drought in both genotypes [51]. Beyond this, the transcript and protein levels of other glutathione S-transferases GSTZ1 (HORVU5Hr1G012160; AT2G02390) or GSTU25 (HORVU5Hr1G103420; AT1G17180) were up-regulated by drought specifically in the single genotype, WT and *hvd14*, respectively. Thus, glutathione metabolism might be part of an SL-dependent defence response, which is disturbed in *hvd14* resulting in enhanced drought sensitivity. Interestingly, since glutathione is involved in ROS scavenging and ABA-induced stomatal closure [52], modulation of glutathione metabolism may be one of the crucial mechanisms underlying barley drought response.

### Dehydrins and water transport

Beyond ROS scavenging, other antioxidative cell mechanisms are related to increasing protein stability and preventing aggregation. The primary players in these processes are the dehydrins [53], a group of late embryogenesis abundant (LEA) proteins. Dehydrins are involved in plant response to various stresses, including drought, frost, and salinity [54]. Moreover, DEHYDRIN 5 (DHN5) was induced by drought via the ABA-mediated signaling pathway in barley [55]. Among the nine barley dehydrins, the transcriptional expression of nine and six were up-regulated by drought in WT and *hvd14*, respectively*.* Within those six common dehydrins, the gene HORVU5Hr1G092120 was one of the top 10 up-regulated genes induced by drought in both genotypes. However, three dehydrin genes (HORVU5Hr1G092160, HORVU5Hr1G092100 / HORVU5Hr1G092150—AT3G50980 and HORVU6Hr1G083960—AT1G20440) were strongly induced by drought in WT only (Supplemental Data 1, Supplemental Data 8). Analysis of their promoter regions (1500 bp) revealed that two of three remain under the regulation of three SL-dependent TFs previously identified (HORVU4Hr1G023110, HORVU1Hr1G060490, HORVU6Hr1G092750—AT5G04390) (Supplemental Data 8). Interestingly the expression of one of the dehydrins (HORVU6Hr1G064620) was up-regulated by drought in both genotypes. Additionally, the higher abundance of the protein encoded by this gene was noted for WT and *hvd14,* in response to drought (Supplemental Data 8). Collectively, this data indicates that these TFs may regulate dehydrin expression in conjunction in an SL-dependent manner, whereas some dehydrins (e.g. HORVU6Hr1G064620) are drought-induced independently of SLs.

Aquaporins belong to the intrinsic membrane proteins, which are involved in the passive movement of water and other substrates, including ROS, across the plasma membranes (PIP) and tonoplast membranes (TIP) [56]. It was shown expression of members of both aquaporin classes was modified by drought in barley [57]. Interestingly, we resolve very clear and concerted transcriptome and proteome down-regulation of multiple ABA-related PIP: HORVU5Hr1G084230 (PIP2;8; AT2G168500, HORVU2Hr1G089940 (PIP2B; AT2G37170) and TIP: HORVU3Hr1G116790 (TIP1;3; AT4G01470) in *hvd14*, emphasizing a relationship between a lack of ABA sensitivity in *hvd14* and required drought responses in an SL-deficient background. These results may be linked to the lower RWC of *hvd14* when exposed to drought. However, open questions remain about whether the observed expression/abundance pattern is the effect of increased drought sensitivity of *hvd14* or the reason for its phenotype.

### Proteases

Intriguingly, we resolved a total of five proteases that show concerted changes in their transcriptome and proteome levels in response to drought, which are all down-regulated in *hvd14* relative to WT. These include two subtilase-family proteases HORVU6Hr1G081850 (AT3G14067) and HORVU1Hr1G089380 (AT4G10550), one Aspartic proteinase HORVU1Hr1G017570 (APA1; AT1G11910) and a two other proteases HORVU3Hr1G096650 (AT3G19340) and HORVU3Hr1G083760 (AT5G67090). Interestingly, subtilases (SBT) are a generally large protease family founds across eukaryotes that have only recently been related to abiotic stress response [58, 59]. In barley, there are only 11 SBT-related genes [60]. Here, we observed two specifically down-regulated in *hvd14* plants in response to drought, suggesting a novel connection between SL, protein turnover, and nutrient recycling in plants. Interestingly, over-expression of the aspartic acid protease APA1 in Arabidopsis also conveyed drought tolerance by reducing stomatal conductance and water loss, functioning through ABA-dependent signaling [61], and supporting the notion of ABA insensitivity in *hvd14* plants.

## Conclusions

Here we provide insights into the molecular mechanisms underlying the drought-sensitive phenotype of barley SL-insensitive mutant. Using the *hvd14* mutant allowed us to uncover a series of unique SL-dependent genes that have a putative role in barley drought response through managing a wide range of molecular processes. Our findings highlight the need for future research to elucidate the precise role of SLs in regulating these processes via studies on higher-level controllers, including transcription factors, under both normal and drought-stress conditions.

## Methods

### Plant material, growth conditions and phytohormone treatment

Mutant *hvd14.d* was induced by chemical mutagenesis [62]. Drought stress was applied according to the previously described protocol [44]. The survival rate of both genotypes was calculated as described in [44], with some modifications: in the single pot, 15 seedlings of each genotype were placed in two rows (1 genotype – 1 row), at 10 DAS watering was withheld for 15 days, and resumed for three additional days. Thus, the survival rate was calculated at 28 DAS for 120 plants per genotype grown in 8 pots. A paired Student's t-test was applied to check the statistically significant difference between genotypes.

To investigate the effect of SL treatment on barley branching GR24^5DS^ (StrigoLab, Turin, Italy) was used. Four seeds of each genotype were sown in pots (7.5 × 7.5 × 10 cm) filled with garden soil and watered every day with 50 ml (1–10 DAS) or 100 ml (11–18 DAS) with 10 µM of GR24^5DS^ or 0.01% of acetone (control). A paired Student's t-test was applied to check the statistically significant difference between samples. For each treatment, 32 plants grown in eight pots were analyzed.

### Tissue collection

To conduct RNA-seq analyses, we gathered plant tissue in four separate biological replicates. Each replicate included 2 cm-long sections of the second leaf, positioned 3 cm below the leaf tip. These sections were taken from both control and drought-stressed plants of both genotypes. We utilized the same type of tissue for SEM analysis, measuring cytosine methylation, and conducting DAB staining. For proteome analysis and measuring phytohormone levels, we collected entire shoots from both control and drought-stressed plants. This collection also consisted of four biological replicates, each containing four plants.

### Transcriptomic analysis

Transcriptomic analysis were performed as described previously [44]. cDNA libraries were prepared for four biological replicates for each condition (C – control, D—drought) for each genotype (WT and *hvd14* mutant).

### Proteomic analysis

Total protein extracts were prepared from frozen ground tissue using an SDS-lysis buffer (4% SDS, 50 mM HEPES–KOH, pH 8.0). Samples were clarified by centrifuging at 20,000 × g for 15 min at room temperature. After quantification using a BCA assay (ThermoScientific, 23,225), 500 µg of protein from each sample was aliquoted for processing. These samples were reduced with 10 mM dithiothreitol (DTT) at 95 °C for five minutes, cooled, and alkylated by incubation with 30 mM iodoacetamide for 30 min in the dark. Iodoacetamide was quenched by further addition of 10 mM of DTT. Samples were then prepared for trypsin digestion using a manual version of the R2-P1 protocol [63]. Briefly, proteins were bound to carboxylated magnetic beads, washed with 80% (v/v) ethanol to remove SDS, and then mixed with a solution of trypsin (at a 1:100 trypsin to protein ratio) (Sequencing Grade Modified Trypsin; Promega V5113). The digestion reaction was performed at 37 °C overnight in a shaking incubator at 150 rpm. Digested peptides were eluted in water and desalted using ZipTips (MilliporeSigma, ZTC18S008) according to the manufacturer's protocol.

Peptides were analysed on a Orbitrap Fusion Lumos Tribrid Orbitrap mass-spectrometer, 1 µg of peptides were injected using an Easy-nLC 1200 system (ThermoScientific) and separated on a 50 cm Easy-Spray Pep-Map column (ES803A; ThermoScientific). Peptides were eluted with a 120 min linear solvent B (0.1% Formic Acid in 80% acetonitrile) gradient (4%—41% B) with an additional 5 min step (41%-98%). The acquisition was performed in data-dependent mode using the Universal Method (ThermoScientific). Full-scan MS^1^ spectra (350–2000 m/z) were acquired in the Orbitrap at a resolution of 120,000 with a normalized AGC target of 125% and a maximum injection time of 100 ms. MS^2^ was acquired in the ion-trap using quadrupole isolation in a window of 2.5 m/z with dynamic exclusion for 30 s. Selected ions were HCD fragmented with 35% fragmentation energy, an AGC target of 200% and a maximum injection time of 100 ms.

Raw mass-spec files were processed using MaxQuant software version 1.6.14 [64]. Spectra were searched against a custom-made decoyed (reversed) version of the barley proteome from the r1 IBSC genome assembly (Phytozome genome ID: 462). Trypsin specificity was set to two missed cleavages and a protein and PSM false discovery rate of 1% each was applied. The minimum peptide length was set to seven and match between runs was enabled. Fixed modifications included cysteine carbamidomethylation and variable modifications included methionine oxidation. MaxQuant results were then processed using Perseus version 1.6.14.0 [65]. Reverse hits and contaminants were removed, data was log-transformed and filtered by applying a threshold of valid quant values in at least 2 of 3 replicates in at least one experimental group. Missing values were imputed from a normal distribution and significantly changing differentially abundant proteins were determined using a Benjamini–Hochberg corrected *p–value* threshold of < 0.05.

### Transcriptome and proteome association network

Arabidopsis homologs for all barley genes exhibiting a significant change in transcript- (Log_2_FC > 1.5; *p-value* < 0.01) and protein abundance (Log_2_FC > 0.58; *q-value* < 0.05) were obtained using BioMart tool (https://plants.ensembl.org/biomart) from `Arabidopsis thaliana genes (TAIR10)` dataset. The association network was constructed in Cytoscape (v. 3.9) using a combination of the string-db and enhancedGraphics plug-ins and a stringdb edge score of > 0.4. Color scale depicts Log_2_ fold-change in abundance. Log_2_FC > 1.5 threshold was used for transcriptomic data to ensure the readability of data visualization by reducing the number of DEGs to those with the largest fold change.

### Phytohormone measurements

Previously described Multiple Phytohormone Profling by Targeted Metabolomics was applied to measure phytohormone content in barley tissue [66]. Three technical replicates were performed for each of two sets of tissue for each genotype and time point. A paired Student's t-test was applied to check the statistically significant difference between samples.

### Cytosine methylation (5mC)

Nuclei suspension for 5mC analysis was prepared as described earlier [67] with minor modifications. Tissue sections from leaves, immunostaining and image acquisition and processing were carried out as previously described [68–70]. Briefly, the following primary mouse monoclonal antibody against methylated DNA: anti-5-methyl-cytosine (1:00, Abcam, Cat. no. ab73938) and secondary antibody: Alexa Fluor 488 goat anti-mouse (Invitrogen, Molecular Probes, Cat. no. A-11001) were used. Images from leaf sections were registered using confocal laser scanning microscopy (CLSM) (Olympus FV1000) and high content screening fluorescence microscopy (HCSFM) (Scan'R, Olympus). Image processing operations were performed with ImageJ (Fiji) or the automated segmentation-based Scan'R Analysis software (Olympus). An average of 100 – 400 nuclei (depending on the method used) were analysed for each experimental group. Alexa 488 fluorescence (5mC) was segmented with the threshold value parameter, and then the fluorescence intensity was measured. The mean fluorescence intensity values were estimated in the relative units and shown in Figs. 3A and B. A paired Student's t-test was applied to check the statistically significant difference between samples.

### SEM analysis

The middle part of the 2^nd^ leaf of a least six different plants was placed in between blotting paper and air dried for ten days in between book pages to avoid possible structural changes of the wax layer caused by alcohol dehydration. Next, 0.5 mm^2^ pieces were attached to aluminium stubs with adhesive carbon tabs (Agar, Scietific, Essex, UK) with the upper or lower epidermis of the leaf facing up. Cross sections were applied laterally with the open-cut surface facing up. Samples were gold-sputtered (Quorum EMS 150R ES Plus, Laughton, UK) and examined at 5 kV in a Zeiss Gemini300 scanning electron microscope (Carl Zeiss Microscopy GmbH, Oberkochen).

### Wax extraction and composition analysis

The wax composition on the leaf surface was analysed after extraction in hot chloroform, as described earlier [32]. To assess statistical significance between samples, a paired Student's t-test was employed.

### DAB staining against hydrogen peroxide

Ten leaf fragments collected from ten separate plants were placed in a single falcon tube filled with a staining solution prepared according to the manufacturer's instruction (DAB Substrate Kit, Thermo Fisher Scientific) with Tween 20 (0.05% v/v). Leaves were incubated for eight hours in darkness at a shaker (80–100 rpm) and then treated with bleaching solution (ethanol: acetic acid: glycerol = 3:1:1) for 20 min at 95 °C. Next, samples were washed with fresh bleaching solution for 30 min and photographed.

### TF prediction and identification of TF binding sites

Amino acid sequences of DEG and DAP were obtained using the Biomart tool (https://plants.ensembl.org/biomart) from `Hordeum vulgare genes (IBSC v2)` dataset. Those sequences were used as a query in the tool TF Prediction from Plant TFDB (http://planttfdb.gao-lab.org/prediction.php). Promoter sequences (1500 bp before START codon) of DEG and DAP were obtained using the Biomart tool (https://plants.ensembl.org/biomart) from `Hordeum vulgare genes (IBSC v2)` dataset. Those sequences were used as a query in the tool Binding Site Prediction from Plant TFDB (http://plantregmap.gao-lab.org/binding_site_prediction.php) against *Hordeum vulgare* database with threshold *p-value* ≤ 1e^−4^. Obtained MLOC IDs were translated to HORVU ID via blasting amino acid sequences of MLOC against barley HC Proteins IBSC_v1 dataset using Galaxy server (https://galaxy-web.ipk-gatersleben.de/).

### Supplementary Information

**Additional file 1: Supplemental Figures, Supplemental Figure 1. **Treatment with synthetic SL analogue (GR245DS) confirmed SL-insensitivity of hvd14. A. 18-day-old seedlings of both genotypes treated with 10 µM GR245DS or control solution (0.01% acetone). B. Effect of GR245DS treatment on barley branching. Bars represent the mean ±SE (*n*=32). Asterisks indicate significant differences as determined by Student’s t-test (***P≤0.001).  **Supplemental Figure 2.** Plant survival of WT and hvd14. A. Overview of the experimental setup: in the single pot 15 seedlings of each genotype were grown together. B. The phenotype of plants after 15 days without watering and three additional days with re-watering. C. Plant survival of both genotypes. Bars represent the mean ±SE (*n*=120). Asterisks indicate significant differences as determined by Student’s t-test (***P≤0.001). **Supplemental Figure 3.** Overview of differentially expressed genes (DEGs) and differentially expressed proteins (DEPs) identified in the present work. **Supplemental Figure 4.** Phytohormone-related genes and proteins differentially expressed in WT and hvd14 mutant, in response to drought. A. Number of ABA-related genes identified in both genotypes. B. Number of JA-related genes identified in both genotypes. C. Transcriptome and proteome data for compounds involved in JA biosynthesis, which were regulated by drought in both genotypes. D. Transcriptome and proteome data for compounds involved in CK signalling, which were regulated by drought in both genotypes.

**Additional file 2:** **Supplemental Data 1. Table S1a. **List of up-regulated genes  in the WT_D vs WT_C comparison (log2FC ≥ 1, adjusted P value ≤ 0.01). **Table S1b.** List of down-regulated genes  in the WT_D vs WT_C comparison (log2FC ≤ -1, adjusted P value ≤ 0.01). **Table S1c.** List of up-regulated genes  in the hvd14_D vs hvd14_C comparison (log2FC ≥ 1, adjusted P value ≤ 0.01). **Table S1d. **List of down-regulated genes  in the hvd14_D vs hvd14_C comparison (log2FC ≤ -1, adjusted P value ≤ 0.01). **Table S1e. **List of genes up-regulated by drought, only in WT (log2FC ≥ 1, adjusted P value ≤ 0.01) - WT Drought Resposne Specific (WT DRS) genes. **Table S1f. **List of genes down-regulated by drought, only in WT (log2FC ≤ -1, adjusted P value ≤ 0.01) - WT Drought Resposne Specific (WT DRS) genes. **Table S1g. **List of genes up-regulated by drought, only in hvd14 (log2FC ≥ 1, adjusted P value ≤ 0.01) - hvd14 Drought Resposne Specific (hvd14 DRS) genes. **Table S1h. **List of genes down-regulated by drought, only in hvd14 (log2FC ≤ -1, adjusted P value ≤ 0.01) - hvd14 Drought Resposne Specific (hvd14 DRS) genes. **Table S1i. **List of genes up-regulated by drought, in both genotypes: WT and hvd14 (log2FC ≤ -1, adjusted P value ≤ 0.01).  **Table S1j. **List of genes down-regulated by drought, in both genotypes: WT and hvd14 (log2FC ≤ -1, adjusted P value ≤ 0.01).

**Additional file 3: Supplemental Data 2. Table S2a. **List of up-regulated proteins in the WT_D vs WT_C comparison (log2FC ≥ 0.58, q-value ≤ 0.05). **Table S2b. **List of down-regulated proteins in the WT_D vs WT_C comparison (log2FC ≤ -0.58, q-value ≤ 0.05). **Table S2c. **List of up-regulated proteins in the hvd14_D vs hvd14_C comparison (log2FC ≥ 0.58, q-value ≤ 0.05). **Table S2d. **List of down-regulated proteins in the hvd14_D vs hvd14_C comparison (log2FC ≤ -0.58, q-value ≤ 0.05). **Table S2e. **List of proteins up-regulated by drought, only in WT (log2FC ≥ 0.58, q-value ≤ 0.05) - WT Drought Resposne Specific (WT DRS) proteins. **Table S2f. **List of proteins down-regulated by drought, only in WT (log2FC ≤ 0.58, q-value ≤ 0.05) - WT Drought Resposne Specific (WT DRS) proteins. **Table S2g. **List of proteins up-regulated by drought, only in hvd14 (log2FC ≥ 0.58, q-value ≤ 0.05) - hvd14 Drought Resposne Specific (hvd14 DRS) proteins. **Table S2h. **List of proteins down-regulated by drought, only in hvd14 (log2FC ≤ 0.58, q-value ≤ 0.05) - hvd14 Drought Resposne Specific (hvd14 DRS) proteins. **Table S2i. **List of proteins up-regulated by drought, in both genotypes: WT and hvd14 (log2FC ≥ 0.58, q-value ≤ 0.05). **Table S2j. **List of proteins down-regulated by drought, in both genotypes: WT and hvd14 (log2FC ≥ 0.58, q-value ≤ 0.05).

**Additional file 4: Supplemental Data 3. **A list of Arabidopsis homologs for all barley genes exhibiting a significant change in transcript- and protein abundance which were used for the creation of an association network presented in Figure 3.

**Additional file 5: Supplemental Data 4. **TFs identified among DEG specific for WT.

**Additional file 6: Supplemental Data 5. Table S5a. **TF binding sites in the promoter region (1500 bp) of WT DRS up-regulated genes. **Table S5b. **TF binding sites in the promoter region (1500 bp) of WT DRS down-regulated genes. **Table S5c. **TF binding sites in the promoter region (1500 bp) of WT DRS up-regulated proteins. **Table S5d. **TF binding sites in the promoter region (1500 bp) of WT DRS down-regulated proteins.

**Additional file 7: Supplemental Data 6. **Gene Ontology enrichment for DEG and DAP, which remain under the control of identified TFs.

**Additional file 8: Supplemental Data 7. Table S7a. **Expression of genes described as crucial in wax biosynthesis (Daszkowska-Golec et al., 2020) or annotated to the ‘cutin biosynthetic process’ and ‘cuticle development’ induced by drought in WT and/or hvd14. **Table S7b. **The analyses of epicuticular waxes in WT and hvd14 under control (C) and drought (D) conditions (µg/g dry weight).

**Additional file 9: Supplemental Data 8. Table S8a. **Transcriptome and proteome response of dehydrins to drought stress. **Table S8b.** Number of TF binding sites in the promoter of genes encoding dehydrins. 

## Data Availability

Transcriptomic data: - E-MTAB-12804: https://www.ebi.ac.uk/biostudies/arrayexpress/studies/E-MTAB-12804 - E-MTAB-12796: https://www.ebi.ac.uk/biostudies/arrayexpress/studies/E-MTAB-12796 Proteomic data: - PXD040828: https://www.ebi.ac.uk/pride/archive/projects/PXD040828

## Abbreviations

5mC: 5-Methylcytosine
ABA: Abscisic acid
C: Control
CK: Cytokinin
CLSM: Confocal laser scanning microscopy
D: Drought
DAP: Differentially abundant proteins
DAS: Days after sowing
DEG: Differentially expressed genes
DR: Drought response
DRS: Drought response specific
FC: Fold change
HCSFM: High content screening fluorescence microscopy
JA: Jasmonic acid
PA: Phaseic acid
ROS: Reactive oxygen species
RWC: Relative water content
SEM: Scanning electron microscope
SLs: Strigolactones
TF: Transcription factor
WT: Wild type
