# Supplementary material for: Multi-omics insights into the positive role of strigolactone perception in barley drought response
Source: BMC Plant Biol. 2023 Sep 22;23:445. doi: 10.1186/s12870-023-04450-1 (PMC10515045; doi:10.1186/s12870-023-04450-1)
Supplement: Supplementary file 1 — Additional file 1: Supplemental Figures, Supplemental Figure 1. Treatment with synthetic SL analogue (GR245DS) confirmed SL-insensitivity of hvd14. A. 18-day-old seedlings of both genotypes treated with 10 µM GR245DS or control solution (0.01% acetone). B. Effect of GR245DS treatment on barley branching. Bars represent the mean ±SE (n=32). Asterisks indicate significant differences as determined by Student’s t-test (***P≤0.001). Supplemental Figure 2. Plant survival of WT and hvd14. A. Overview of the experimental setup: in the single pot 15 seedlings of each genotype were grown together. B. The phenotype of plants after 15 days without watering and three additional days with re-watering. C. Plant survival of both genotypes. Bars represent the mean ±SE (n=120). Asterisks indicate significant differences as determined by Student’s t-test (***P≤0.001). Supplemental Figure 3. Overview of differentially expressed genes (DEGs) and differentially expressed proteins (DEPs) identified in the present work. Supplemental Figure 4. Phytohormone-related genes and proteins differentially expressed in WT and hvd14 mutant, in response to drought. A. Number of ABA-related genes identified in both genotypes. B. Number of JA-related genes identified in both genotypes. C. Transcriptome and proteome data for compounds involved in JA biosynthesis, which were regulated by drought in both genotypes. D. Transcriptome and proteome data for compounds involved in CK signalling, which were regulated by drought in both genotypes. [file 12870_2023_4450_MOESM1_ESM.pptx]

## Slide 1
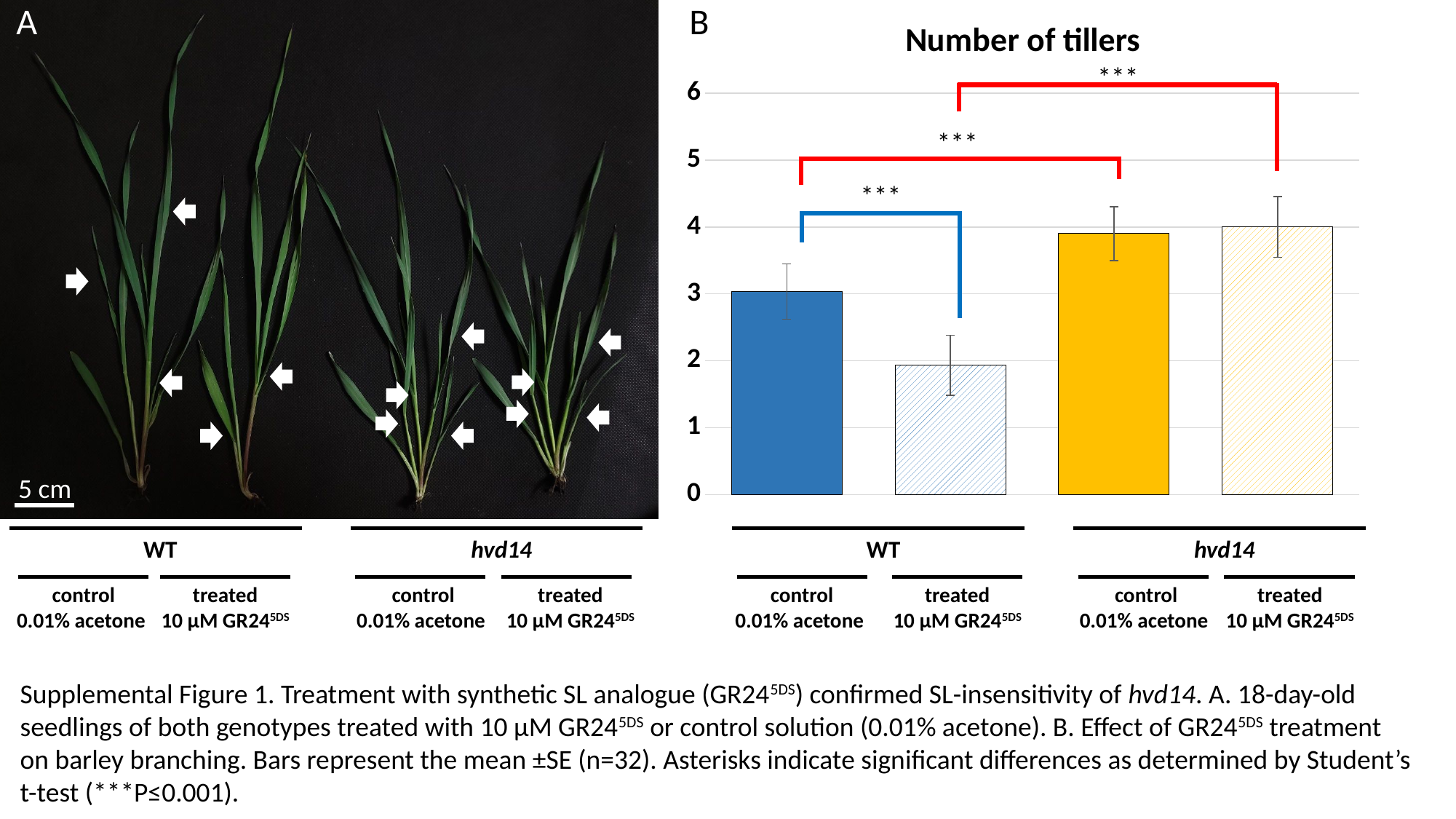

B
A
### Chart: Number of tillers
| Category | |
|---|---|
| WT | 3.033333333333333 |
| WT GR24 | 1.9333333333333333 |
| hvd14 | 3.9 |
| hvd14 GR24 | 4.0 |***
***
5 cm
WT
hvd14
WT
hvd14
control
0.01% acetone
treated
10 µM GR245DS
control
0.01% acetone
treated
10 µM GR245DS
control
0.01% acetone
treated
10 µM GR245DS
control
0.01% acetone
treated
10 µM GR245DS
Supplemental Figure 1. Treatment with synthetic SL analogue (GR245DS) confirmed SL-insensitivity of hvd14. A. 18-day-old seedlings of both genotypes treated with 10 µM GR245DS or control solution (0.01% acetone). B. Effect of GR245DS treatment on barley branching. Bars represent the mean ±SE (n=32). Asterisks indicate significant differences as determined by Student’s t-test (***P≤0.001).

## Slide 2
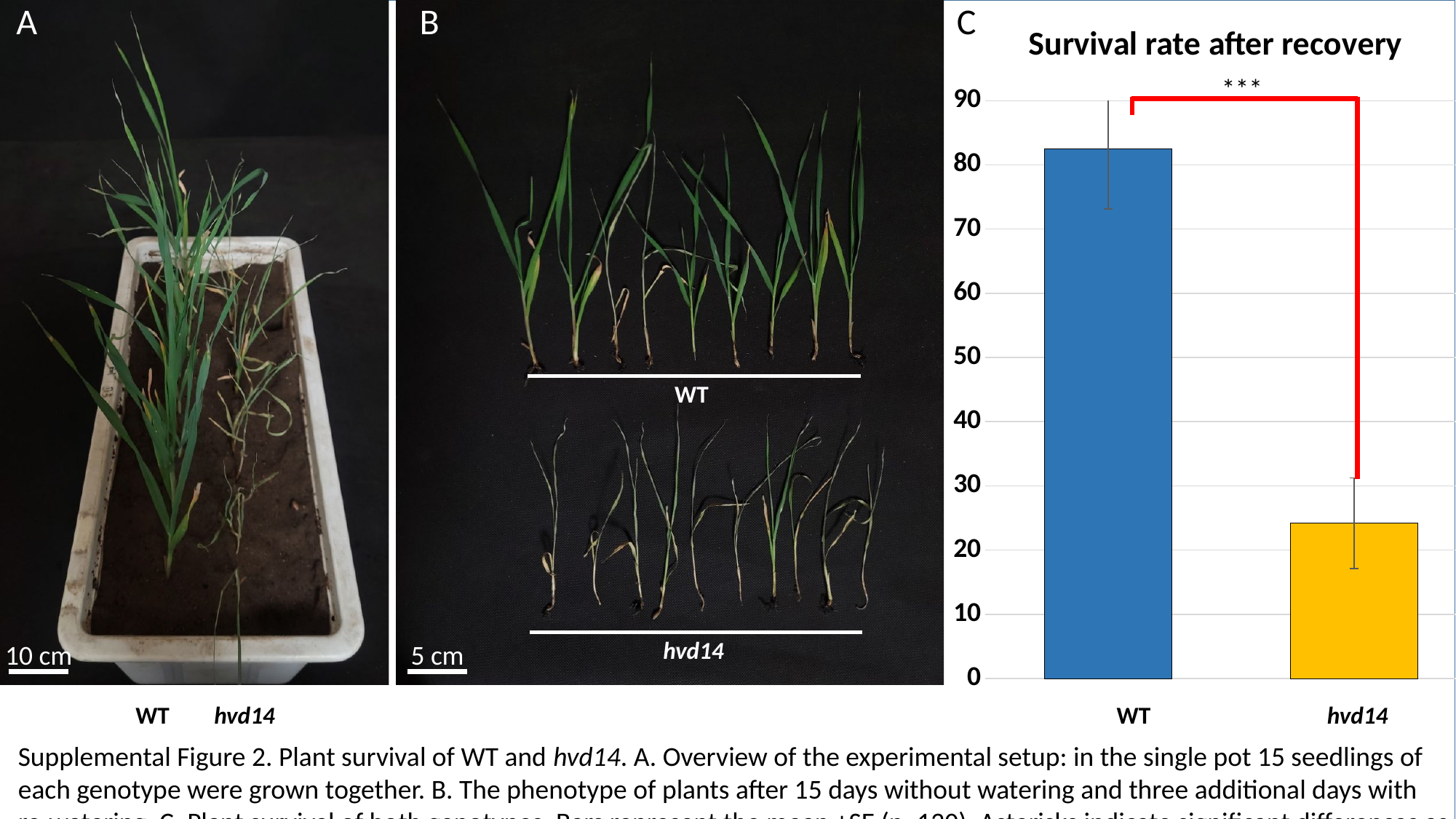

### Chart: Survival rate after recovery
| Category | |
|---|---|
| WT | 82.49999999999999 |
| hvd14 | 24.166666666666668 |B
C
A
***
WT
hvd14
10 cm
5 cm
WT
hvd14
WT
hvd14
Supplemental Figure 2. Plant survival of WT and hvd14. A. Overview of the experimental setup: in the single pot 15 seedlings of each genotype were grown together. B. The phenotype of plants after 15 days without watering and three additional days with re-watering. C. Plant survival of both genotypes. Bars represent the mean ±SE (n=120). Asterisks indicate significant differences as determined by Student’s t-test (***P≤0.001).

## Slide 3
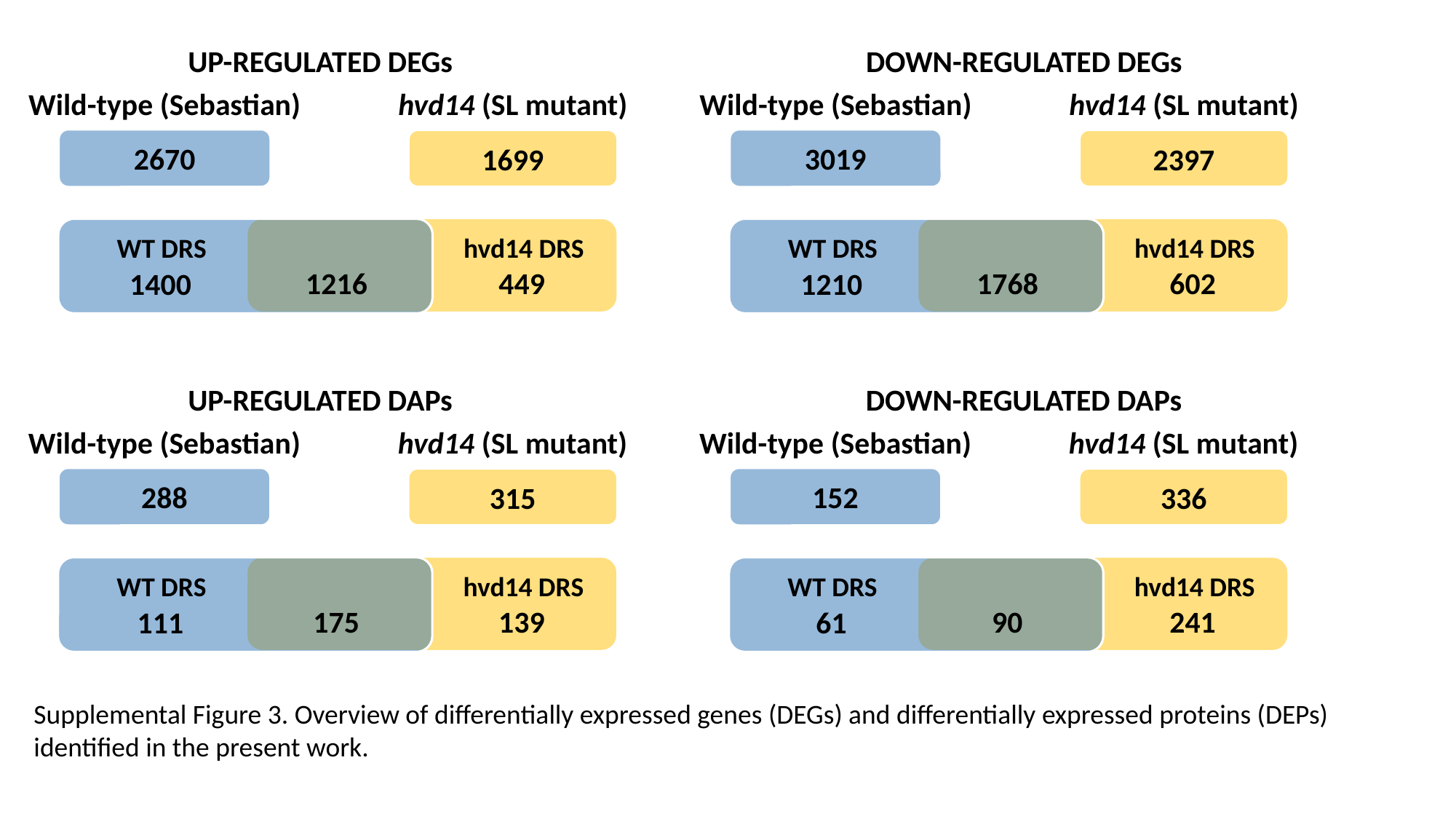

UP-REGULATED DEGs
DOWN-REGULATED DEGs
hvd14 (SL mutant)
hvd14 (SL mutant)
Wild-type (Sebastian)
Wild-type (Sebastian)
2670
3019
1699
2397
WT DRS
hvd14 DRS
WT DRS
hvd14 DRS
1216
1768
449
602
1400
1210
UP-REGULATED DAPs
DOWN-REGULATED DAPs
hvd14 (SL mutant)
hvd14 (SL mutant)
Wild-type (Sebastian)
Wild-type (Sebastian)
288
152
315
336
WT DRS
hvd14 DRS
WT DRS
hvd14 DRS
175
90
139
241
111
61
Supplemental Figure 3. Overview of differentially expressed genes (DEGs) and differentially expressed proteins (DEPs) identified in the present work.

## Slide 4
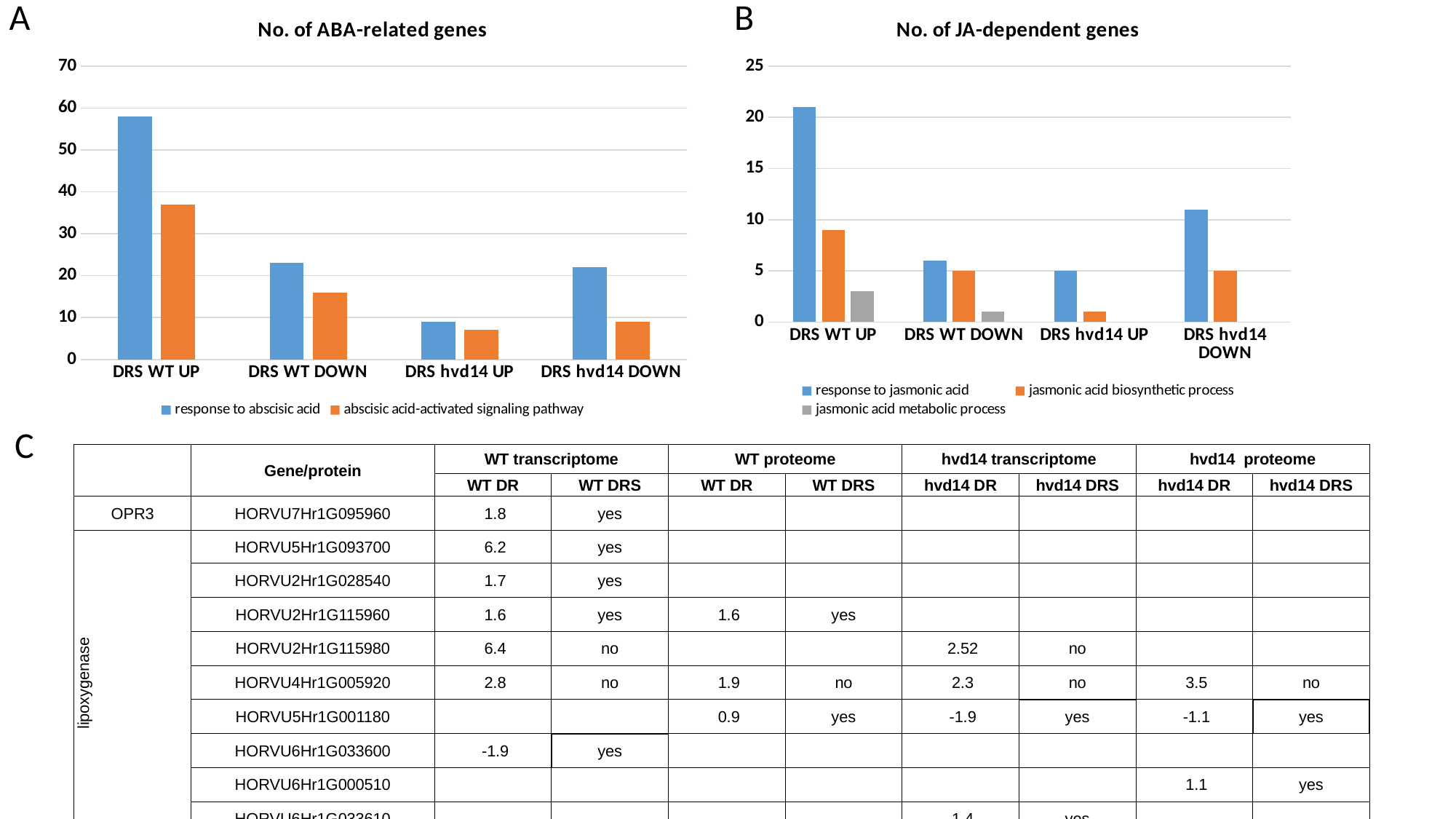

### Chart: No. of ABA-related genes
| Category | response to abscisic acid | abscisic acid-activated signaling pathway |
|---|---|---|
| DRS WT UP | 58.0 | 37.0 |
| DRS WT DOWN | 23.0 | 16.0 |
| DRS hvd14 UP | 9.0 | 7.0 |
| DRS hvd14 DOWN | 22.0 | 9.0 |A
B
### Chart: No. of JA-dependent genes
| Category | response to jasmonic acid | jasmonic acid biosynthetic process | jasmonic acid metabolic process |
|---|---|---|---|
| DRS WT UP | 21.0 | 9.0 | 3.0 |
| DRS WT DOWN | 6.0 | 5.0 | 1.0 |
| DRS hvd14 UP | 5.0 | 1.0 | 0.0 |
| DRS hvd14 DOWN | 11.0 | 5.0 | 0.0 |Supplemental Figure 4. Phytohormone-related genes and proteins differentially expressed in WT and hvd14 mutant, in response to drought. A. Number of ABA-related genes identified in both genotypes. B. Number of JA-related genes identified in both genotypes. C. Transcriptome and proteome data for compounds involved in JA biosynthesis, which were regulated by drought in both genotypes. D. Transcriptome and proteome data for compounds involved in CK signalling, which were regulated by drought in both genotypes.
C
| | Gene/protein | WT transcriptome | | WT proteome | | hvd14 transcriptome | | hvd14 proteome | |
| --- | --- | --- | --- | --- | --- | --- | --- | --- | --- |
| | | WT DR | WT DRS | WT DR | WT DRS | hvd14 DR | hvd14 DRS | hvd14 DR | hvd14 DRS |
| OPR3 | HORVU7Hr1G095960 | 1.8 | yes | | | | | | |
| lipoxygenase | HORVU5Hr1G093700 | 6.2 | yes | | | | | | |
| | HORVU2Hr1G028540 | 1.7 | yes | | | | | | |
| | HORVU2Hr1G115960 | 1.6 | yes | 1.6 | yes | | | | |
| | HORVU2Hr1G115980 | 6.4 | no | | | 2.52 | no | | |
| | HORVU4Hr1G005920 | 2.8 | no | 1.9 | no | 2.3 | no | 3.5 | no |
| | HORVU5Hr1G001180 | | | 0.9 | yes | -1.9 | yes | -1.1 | yes |
| | HORVU6Hr1G033600 | -1.9 | yes | | | | | | |
| | HORVU6Hr1G000510 | | | | | | | 1.1 | yes |
| | HORVU6Hr1G033610 | | | | | 1.4 | yes | | |
D
| | Gene/protein | WT transcriptome | | hvd14 transcriptome | | hvd14 proteome | |
| --- | --- | --- | --- | --- | --- | --- | --- |
| | | WT DR | WT DRS | hvd14 DR | hvd14 DRS | hvd14 DR | hvd14 DRS |
| CK activation | HORVU1Hr1G041640 | -1.7 | yes | | | | |
| | HORVU3Hr1G066810 | | | 1.5 | yes | | |
| CK deactivation | HORVU2Hr1G079420 | 9.27 | yes | | | | |
| | HORVU1Hr1G007820 | 2.18 | yes | | | | |
| | HORVU2Hr1G090150 | | | -3.68 | yes | | |
| | HORVU6Hr1G082710 | | | | | -1.05 | yes |
| CK receptor | HORVU7Hr1G111360 | | | 1.04 | yes | | |
Supplemental Figure 4. Phytohormone-related genes and proteins differentially expressed in WT and hvd14 mutant, in response to drought. A. Number of ABA-related genes identified in both genotypes. B. Number of JA-related genes identified in both genotypes. C. Transcriptome and proteome data for compounds involved in JA biosynthesis, which were regulated by drought in both genotypes. D. Transcriptome and proteome data for compounds involved in CK signalling, which were regulated by drought in both genotypes.
